# Effect of caffeine on lipid profile in ciclism practitioners

**DOI:** 10.1186/1550-2783-9-S1-P20

**Published:** 2012-11-19

**Authors:** Antonio Felipe Correa Marangon, Tatiana Helou, Deilys Vazquez Gonzalez

**Affiliations:** 1 Centro Universitario UniCEUB, Brazil

## Background

Caffeine is a β2 agonist that increases energy expenditure both at rest and during sports, increasing lipolysis and fatty acid oxidation. Caffeine may also increase the utilization of lipids as energy source during aerobic exercises.

## Methods

The objective of this study was to investigate if caffeine can influence lipid profile in trained cyclists. 19 trained and familiarized male cyclists with a mean age of 35 ±8.1 were randomly assigned to placebo (n=7) and caffeine groups (n=12). 30 minutes before the exercise each member of the caffeine group received 5mg/Kg of caffeine. All participants underwent the same pre-test meal 2 hours before the test and were in 8 hours of fasting. Trials consisted of 60 min cycling at approximately 70-85% VO_2max_. The study was double blind and a students t test was used for our statistical analysis (*p* values <0.05). Blood samples were collected before and after the test for total cholesterol, LDL-cholesterol, HDL-cholesterol and triglycerides.

## Results

The average total cholesterol, before and after the caffeine group (CG), was 192.83 ±38mg/dL and 212.75 ±48mg/dL, respectively. In the placebo group (PG) the mean total cholesterol was 162.71 ±92mg/dL before and 180.43 ±43mg/dL after. The HDL-cholesterol fraction in the caffeine group before and after was 43.42 ±12mg/dL and 53 ±14mg/dL, respectively. In the placebo group the fraction HDL-cholesterol before was 34.57±8mg/dL and after 42.43 ±11mg/dL. The LDL-cholesterol before and after in the caffeine group was 133.17 ±72mg/dL and 143.5 ±99mg/dL, respectively. In the placebo group LDL-cholesterol before was 108.86±25mg/dL and after 120.14 ±60mg/dL. Finally, the triglycerides in the caffeine group before and after were 81.83±24mg/dL and 81.25 ±29mg/dL, respectively. In the placebo group the triglycerides before were 96.86 ±32mg/dL and after 87.57 ±28mg/dL. There was a significant difference only in the values of total cholesterol (p=0.041) and HDL-cholesterol (p=0.001) between the participants of the caffeine group. Between the groups there was no significant difference (p>0.05) in all lipid markers (total cholesterol p=0.755, triglycerides p=0.560, HDL-cholesterol p=0.951, LDL-cholesterol p=0.836).

## Conclusions

From the results that were found, we can conclude that caffeine doesn’t interfere in the lipid profile in cyclists. In addition one exercise session was capable of increasing the plasmatic levels of HDL-cholesterol. We suggest that other studies should be conducted in order to check for how long the plasmatic levels of HDL-cholesterol remain elevated after cycling exercise.

